# Vertigo due to cerebellar cavernous malformation: A case report

**DOI:** 10.1016/j.radcr.2022.06.088

**Published:** 2022-07-27

**Authors:** Putri Maharani, Hanik Badriyah Hidayati, Shahdevi Nandar Kurniawan

**Affiliations:** a Neurology Department, Faculty of Medicine, Universitas Airlangga, Dr. Soetomo General Hospital, Surabaya, Indonesia; b Neurology Department, Faculty of Medicine, Universitas Brawijaya, Saiful Anwar General Hospital, Malang, Indonesia

**Keywords:** Cavernous malformation, Central vertigo, Cerebellar, Hemorrhage, CNS, Central Nervous System, CCM, Cerebral Cavernous Malformation, MRI, Magnetic Resonance Imaging, DSA, Digital Subtraction Angiography

## Abstract

Central vertigo is a result of vestibular structure dysfunction in the central nervous system. Currently, misdiagnoses between peripheral and central lesions are frequent, and diagnostic testing costs are high. Identifying the characteristics of these 2 conditions is challenging. We can provide better treatment if we can establish a diagnosis earlier. Cerebral cavernous malformation (CCM) at the cerebellum is a cerebellar lesion that causes symptoms of central vertigo. We report a patient, 20th years old, female, with vertigo for 1 month before being admitted. Vertigo was getting worse, and when the patient arrived at our hospital, vertigo was accompanied by headache, right and left abducens nerve palsy, horizontal nystagmus bidirectional, vertical nystagmus, and weakness on the right side of the body. A brain magnetic resonance imaging (MRI) was performed before surgery and shows a lesion suggestive of CCM at the cerebellum with a hemorrhagic component inside and non-communicating hydrocephalus. There is no vascular malformation based on digital subtraction angiography result. MRI is the most sensitive and specific modality for detecting CCM, whereas cerebral angiography rarely detects this malformation. The patient got surgical treatment, with suboccipital decompression procedures and CCM excision. The histopathological results after surgical treatment revealed a cerebral cavernous malformation. Vertigo, headache, double vision, and weakness on the right side of the body were resolved after surgery.

## Introduction

The most common vestibular symptom is vertigo [1], which is defined as a sensation of movement in the body when it is not moving, and is inconsistent with normal head movements [2]. Vestibular symptoms caused by abnormality in the cerebellum or brain stem are categorized as central type. Symptoms originate from the inner ear or the vestibular nerve referred to peripheral type [3]. The central vestibular system is made up of the vestibular nuclei, cerebellum, brainstem, spinal cord, and vestibular cortex [4]. Any lesion to the projection of vestibular nuclei or the vestibular nuclei itself, can cause vertigo and associated nystagmus [5].

Vertigo has an annual incidence about 3.1% and a prevalence of 22.9% [6]. The various causes of vertigo can be challenging to identify and necessitate careful examination [7]. Vertigo has been classified as either central or peripheral based on the pathology of the vestibular symptoms [6]. Cerebellar disorders are a central cause of vestibular dysfunction. Cerebral cavernous malformation (CCM) located at the cerebellum is an example of cerebellar disorder. The anatomic location of the CCM has a direct impact on the patient's chief complaint. CCM are brain and spinal cord vascular malformations with low-flow that are composed of dilated sinusoidal channels surrounded by endothelial cells that lack of interconnected tight junctions [8]. CCM of the brainstem and cerebellum are less common than supratentorial cavernous malformation. CCM epidemiology is about 0.5 percent of the population, with 18 percent on infratentorial location [9]. Disorder in the cerebellum may lead to central vertigo symptoms.

## Case report

**Fig. 1:**
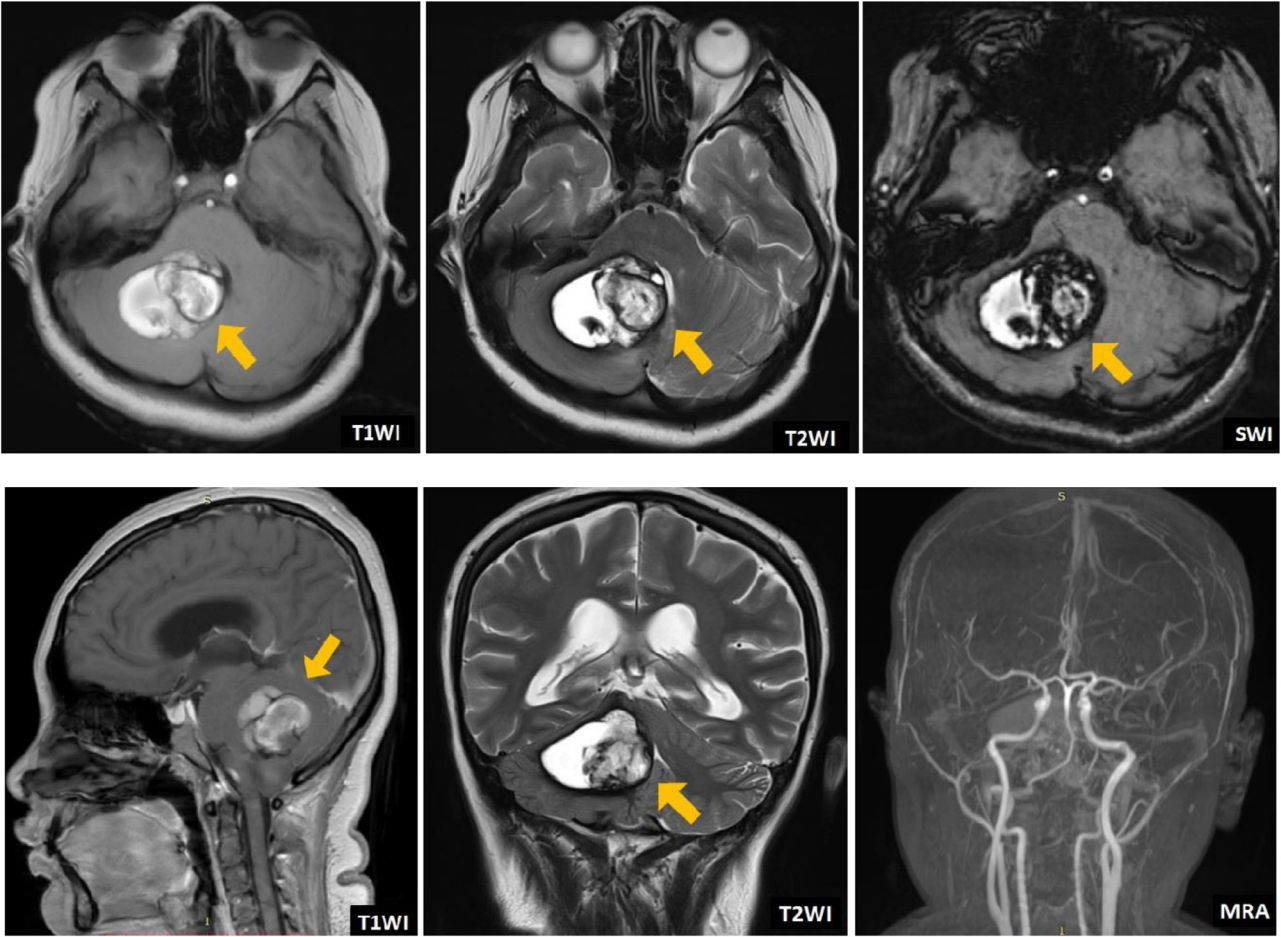
A brain MRI was performed prior to surgery. Yellow arrow in this picture shows the appearance of CCM on axial, coronal and sagittal view. MRA revealed no vascular malformations.

**Fig. 2:**
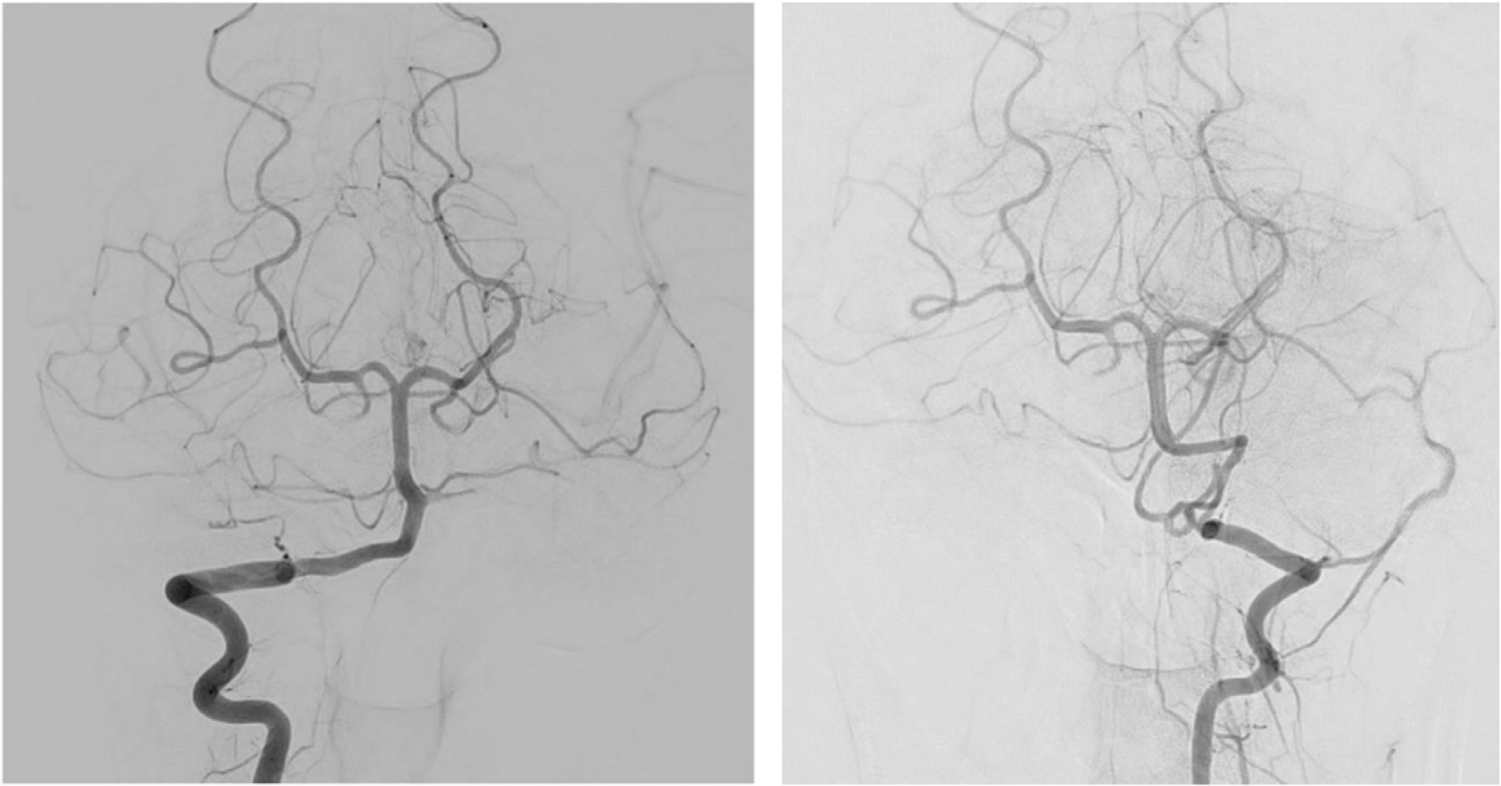
There is no vascular malformation based on digital subtraction angiography.

We report a patient, 20th years old, female, with vertigo since one month before being admitted to our hospital, accompanied by vomit and double vision which get better if she closes one of her eyes. The patient got weakness on the right side of her body and headache on the back side of her head 2 weeks before admission to our hospital. Patient aware, with blood pressure 110/60 mm Hg, heart rate 80 times per minute, temperature 36.7 degrees Celsius, and oxygen saturation 97% with free air. There are right and left abducens palsy, horizontal nystagmus bidirectional, vertical nystagmus, and weakness on the right side of the body. Hematology and blood tests were normal. MRI revealed a lesion suggestive of a cavernous angioma in the cerebellar with a hemorrhagic component inside and non-communicating hydrocephalus. There is a lesion with a hemorrhagic component inside at right cerebellum. The lesion appears hyperintense with hypointense inside on T1W1/T2W1/T2FLAIR, signal drop on SWI, with no contrast enhancement, and compressed aqueduct ventricle, causing narrowing of the ventricle and non-communicating hydrocephalus (Fig. 1). The magnetic resonance angiography showed circle of Willis is patent, vertebral artery hypoplasia (embryonal variant) and there is no vascular malformation. Digital subtraction angiography (DSA) examination remained similar, with no vascular malformations found (Fig. 2).

**Fig. 3:**
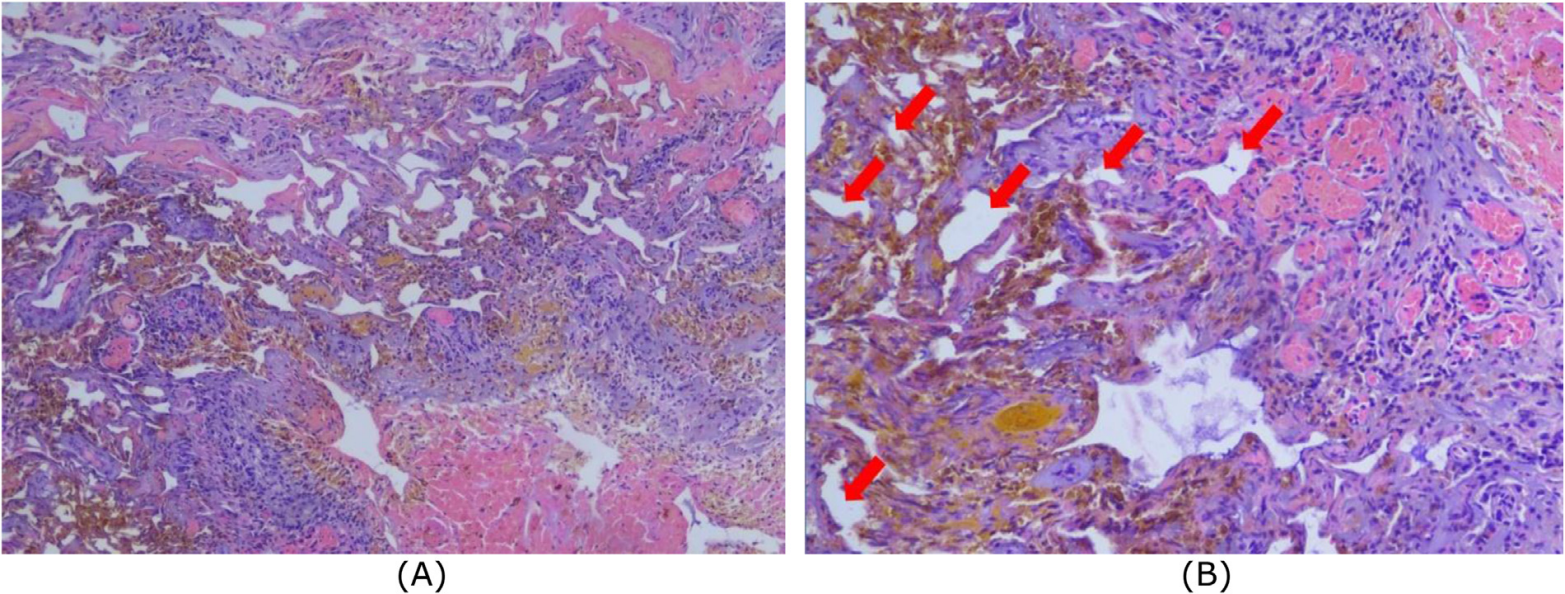
A histopathological examination was performed to confirm the diagnosis of cavernous malformation. Magnificient 100 times (A) and 200 times (B). There is part of brain tissue containing hemorrhages and blood vessels with thin and widened walls, some undergo hyalinization and form sinusoids.

The patient got surgical treatment, the procedures are suboccipital decompression and cavernous angioma excision. The histopathological results after surgical treatment revealed a cavernous angioma, there is part of brain tissue containing hemorrhages and blood vessels with thin and widened walls, some undergo hyalinization and form sinusoids. On the periphery, hemosiderin pigments and infiltration of inflammatory cells of neutrophils and lymphocytes are seen. After surgical treatment dizziness, headache, double vision, and weakness on the right side of her body resolve (Fig. 3).

## Discussion

Peripheral vertigo is responsible for over 90% of all vertigo causes [5]. A life-threatening central cause of acute vestibular syndrome may imitate a less serious peripheral disorder [4]. Based on this, physician especially those in primary care setting or emergency department frequently miss the signs and symptoms of central vertigo. Particularly if the patient is young as in this case report.

Central vertigo is vertigo caused by a central nervous system disease. Injuries to the cerebellum, vestibular nuclei, and their connections within the brainstem caused by hemorrhagic or ischemic events can result in central vertigo. The main features of central vertigo often gradual from week to months, however it can occur suddenly as in stroke. Symptoms of central vertigo less severe than peripheral vertigo, not influenced by position, presence of vertical nystagmus and not fatigable [6]. Disorder in the cerebellum may lead to central vertigo symptoms. In this case, we report a CCM located in the cerebellum.

CCM is also known as cavernous hemangioma, cavernous angioma, or cavernoma [10]. CCM of the brainstem and cerebellum are less common than supratentorial [9]. Cerebellar cavernous malformation accounts for 9.3-52.9% of all infratentorial cases [11]. Horne et al in a recent meta-analysis reported that 6% of CCM were found in the cerebellum [12].

We report our patient, a female, 20th years old with cerebellar cavernous malformation with hemorrhage and non-communicating hydrocephalus. One month prior to admission, the patient complained of vertigo, followed by headache. The patient went to a neurology clinic when the vertigo appeared, and received some pharmacological treatment. The complaints did not improve and she did not return to control in neurology clinic. The symptoms getting worse and the patient was admitted to emergency room, with right and left abducens nerve palsy, horizontal nystagmus bidirectional, vertical nystagmus, and weakness on the right side of the body. Dysmetria, intention tremor, and tonus were difficult to evaluate in the right extremity due to weakness. Neurological deficit in this patient can be caused by hemorrhage at the cerebellum, which compresses the brainstem and ventricle.

In this case, symptoms of central vertigo were found, including a gradual onset and a long duration of vertigo that was not affected by position. There are associated visual symptoms, that is, double vision, and neurological symptoms, that is, weakness on the right side of her body that may be caused by false localizing sign. Nystagmus that appeared is vertical and horizontal nystagmus. Due to signs and symptoms of central vertigo, a CT scan is performed in the emergency room, followed by MRI and DSA to confirm the diagnosis.

It is critical to determining the etiology of central vertigo thorough examination including the Dix-Hallpike test and simple hearing screening test. A comprehensive neurological examination, such as evaluation of ataxia in the extremities and truncal, gait, and also the HINTS test should be done but must be considered the clinical condition of the patient. Every patient suspected of having central vertigo should perform the HINTS test, an abbreviation for head impulse test, nystagmus, and skew deviation. This test is the most effective bedside test for distinguishing peripheral vertigo from central vertigo [5].

In a study by De Oliveira et al., all patients with CCM located in the cerebellum had an acute or sudden onset of headache and cerebellar syndrome components such as ataxia, dysmetria, nystagmus, speech abnormalities, or vomiting [8]. Patients with CCM may present to a health care facility due to a seizure, focal neurologic deficits, accompanied or not with associated hemorrhage, or they may be discovered incidentally when a brain MRI is performed for reasons unrelated to the CCM [13].

If we indicate a central vertigo based on the examination, the patient almost needs to be evaluated further and requires hospitalization. MRI is the modality of choice for detecting a potential infarction, brain tumor, hemorrhage, or demyelination, which can reveal the cause of central vertigo [5]. Because of hemosiderin deposition, CCM typically have a popcorn-like appearance with a low-signal-intensity rim. On T1-weighted images, there is halo of signal hyperintensity surrounds the lesion caused by hemorrhage in subacute stage and blood products degradation [14]. If MRI is not available, computed tomography (CT) may be used [5]. CT is insensitive to small CCM detection but CT scans are widely available and can be used to quickly diagnose acute hematomas, mass effects, and hydrocephalus. When assessing clinical change in CCM patients, a CT scan may be required immediately. However, it is preferable to be followed by an MRI [15]. CCM is better evidence on MRI using specialized techniques like gradient echo or susceptibility-weighted imaging (SWI) [14].

MRI in our case revealed a lesion suggestive of a cavernous angioma in the right cerebellum with a hemorrhagic component inside and non-communicating hydrocephalus. The lesion appears hyperintense with hypointense inside on T1WI/T2WI/T2FLAIR, signal drop on SWI, with no contrast enhancement, and compressed aqueduct ventricle, causing narrowing of the ventricle and non-communicating hydrocephalus. Wu et al [9] discovered that the majority (93.1%) of patients with cerebellar cavernous malformation presented with hemorrhage.

CCM has a lower rupture risk than arteriovenous malformations. The annual hemorrhage rate is estimated to be between 0.7% and 1.1%t [16]. Prior hemorrhage was found to be a risk factor for subsequent hemorrhage, but patient gender, lesion size, location, multiplicity, and radiographically visible associated developmental venous anomalies were not [17].

There is no vascular malformation based on the DSA result from our case report, because of the small size of the abnormal vessels and the extremely low blood flow [16], and thrombi within vascular spaces in CCM [18]. CCM is more difficult to diagnose than other vascular diseases because they are angiographically occult malformations [19]. Angiography can only detect abnormal venous drainage associated with CCM; thus, other imaging techniques are required to provide an accurate diagnosis [19]. According to studies, approximately 20%-85% of cases failed to show abnormal findings, suggesting that angiography has limited efficacy in detection and diagnosis [18].

Because of the clustering of blood-filled caverns, gross specimens of CCM resemble a reddish-purple raspberry. They are normally lobulated and the lesions are well-circumscribed. As a result of the previous hemorrhage, the surrounding cortex is usually hemosiderin stained. Microscopic examination of CCM reveals the common characteristics of vascular sinusoids or "caverns" surrounded by endothelial cells. Inside the caverns, red blood cells are frequently seen. A dense connective-tissue matrix composed of fibroblasts exists between the caverns, is usually present [20]. Pathological anatomy examination after surgical treatment in this case, revealed a CCM, there is part of brain tissue containing hemorrhages and blood vessels with thin and widened walls, some undergo hyalinization and form sinusoids. On the periphery, hemosiderin pigments and infiltration of inflammatory cells of neutrophils and lymphocytes are seen.

CCM treatment options include observation, surgery, and, in rare cases, radiosurgery [13]. The risk of morbidity due to surgery must be considered [9]. Cerebellar cavernous malformation management is equal to those of cerebral cavernous malformation [8]. The patient's clinical presentation affects the treatment plan. Purely incidental CCM is treated conservatively, with yearly MRI scans [19]. Surgery usually results in favorable outcomes for patients with cerebellar cavernous malformation. Early hematoma evacuation may benefit for symptom alleviation and relief of a local mass effect than from actual lesion removal. Patients with severe hemorrhage and unstable clinical conditions may require emergency surgery. It has also been suggested to wait 4-6 weeks after a hemorrhage [11]. Lesion remnants can cause hemorrhaging or seizures, a complete removal is required during surgery [8]. Radiosurgery, particularly Gamma Knife, is an important alternative for treating deep and eloquently located CCM and for patients who do not accept surgical treatment [21].

We report a surgical treatment, with suboccipital decompression procedures and CCM excision for our patient. Indications for surgery include bleeding, neurological deficits, and non-communicating hydrocephalus due to compression of the mass to the ventricles. After surgical treatment, vertigo, headache, double vision, and weakness on the right side of her body resolve.

## Conclusion

Peripheral vertigo has clinical symptoms similar to central vertigo, and it is very important to distinguish between these 2 conditions. Based on history and physical examination performed, we should be able to determine whether the abnormality is a result of a central or peripheral lesion, to consider appropriate examinations and therapy. We report a patient with vertigo caused by CCM at the cerebellum. Location of the CCM has a direct impact on the chief complaint of a patient. Manifestations of CCM in the cerebellum are dizziness, headache, and symptoms of a cerebellar lesion, include: ataxia, dysmetria, nystagmus, abnormalities in speech, or vomiting. CCM located at the cerebellum is a rare case than the supratentorial location. MRI is the modality which is most sensitive and specific for detecting CCM, while cerebral angiography rarely found this malformation. Surgery is a treatment options of CCM and the clinical manifestation, site of the lesion, the number of hemorrhages, as well as the medical comorbidities should be weighed when deciding on treatment strategies.
